# Supplementary material for: The structure of the proteins of Camp Hill virus
Source: PLoS One. 2025 Oct 22;20(10):e0331836. doi: 10.1371/journal.pone.0331836 (PMC12543201; doi:10.1371/journal.pone.0331836)
Supplement: Attachment — Submitted filename: Reviewers comments.doc [file pone.0331836.s001.doc]

**Reviewer: 4**

**Comments to the Author**

In the current manuscript, Thomas et al, presents a structural and immunoinformatic analysis of the proteins of Camp Hill virus (CHV), a newly identified henipavirus in North America. The subject is timely and relevant, given the zoonotic potential of henipaviruses. The integration of structural prediction tools and epitope mapping offers a valuable contribution to early vaccine design efforts. However, the manuscript requires substantial revisions in terms of **scientific rigor, clarity, and novelty justification.**

**Major comments :-**

1. While the identification of CHV is interesting, the manuscript fails to clearly define the **novel scientific insights** derived from this study. It is largely descriptive, with little comparative or functional interpretation
2. The structural analyses and epitope mapping are standard computational exercises. Without **experimental validation,** it is difficult to judge the translational potential
3. The study heavily depends on AlphaFold and I-TASSER predictions. **Confidence metrics (e.g., low C-scores)** suggest many models are unreliable
4. Epitope predictions (IEDB) are speculative without immunological assays or population coverage assessment.
5. No investigation is made into how the predicted epitopes might be processed or presented in the context of actual MHC molecules or host immune systems.
6. The manuscript has several grammatical errors, awkward phrasing (e.g., “CHV is not infected in humans”), and repetitive expressions (e.g., “The predicted structure provides insights…” is repeated verbatim)
7. Figures and tables are cited inconsistently and sometimes lack proper legends or interpretation.
8. The discussion repeats large portions of the introduction
9. A critical appraisal of results is missing, such as **why certain proteins share higher identity with other henipaviruses**, and **implications for cross-reactivity or zoonotic potential**

**Minor comments:-**

1. **Table 3** should include a clearer explanation of how similarity scores were calculated (e.g., pairwise BLAST or Clustal Omega?)
2. **Figures 1–8:** Consider integrating the B-factor and solvent accessibility plots into a single composite figure per protein
3. The **Data Availability Statement** should specify if model PDB files will be made public
4. Abbreviations should be defined consistently on first use (e.g., RMSD, TM-score). Consider preparing an abbreviation table.
