# Supplementary material for: The structure of the proteins of Camp Hill virus
Source: PLoS One. 2025 Oct 22;20(10):e0331836. doi: 10.1371/journal.pone.0331836 (PMC12543201; doi:10.1371/journal.pone.0331836)
Supplement: Attachment — Submitted filename: Response to Reviewers.docx [file pone.0331836.s003.docx]

**Review Comments to the Author**

Please use the space provided to explain your answers to the questions above. You may also include additional comments for the author, including concerns about dual publication, research ethics, or publication ethics. (Please upload your review as an attachment if it exceeds 20,000 characters)

**Reviewer #1:** Reviewer:
Comments to the Author
In the current manuscript, Thomas et al, presents a structural and immunoinformatic analysis of the proteins of Camp Hill virus (CHV), a newly identified henipavirus in North America. The subject is timely and relevant, given the zoonotic potential of henipaviruses. The integration of structural prediction tools and epitope mapping offers a valuable contribution to early vaccine design efforts. However, the manuscript requires substantial revisions in terms of scientific rigor, clarity, and novelty justification.
Major comments :-
1. While the identification of CHV is interesting, the manuscript fails to clearly define the novel scientific insights derived from this study. It is largely descriptive, with little comparative or functional interpretation.

Re: The objective of this manuscript is to define the structure of the proteins of Camp Hill virus. Our study represents the first detailed structural analysis of CHV proteins, using computational analysis, and provides foundational data that will enable future functional and mechanistic studies. While we recognize the value of comparative or functional insights, such analyses—especially those requiring experimental validation—are outside the scope of the present work.

2. The structural analyses and epitope mapping are standard computational exercises. Without experimental validation, it is difficult to judge the translational potential.

Re: In our study, we employed a bioinformatics-driven approach to investigate the structural and immunological features of Camp Hill virus proteins. By computationally modeling these proteins and predicting B-cell and T-cell epitopes, we aimed to lay the groundwork for future experimental research. Although our current work is purely in silico, it serves as a foundational step toward guiding experimental design and prioritizing targets for vaccine or therapeutic development. Ultimately, we recognize that translating computational predictions into practical applications requires rigorous laboratory validation. Our study should therefore be viewed as an initial phase in a broader research pipeline, intended to inform and catalyze further empirical investigation.

3. The study heavily depends on AlphaFold and I-TASSER predictions. Confidence metrics (e.g., low C-scores) suggest many models are unreliable

Re: Current bioinformatic analysis using AlphaFold and I-Tasser have limitations. It is important to interpret these predictions with caution, especially when the models are used to infer functional or immunological characteristics. Structural uncertainty can significantly impact downstream analyses such as active site identification or epitope mapping, potentially leading to misleading conclusions. Therefore, while these computational tools are valuable for hypothesis generation and preliminary insights, experimental validation remains essential to confirm the structural integrity and biological relevance of the predicted models.

4. Epitope predictions (IEDB) are speculative without immunological assays or population coverage assessment.

Re: Epitope mapping is the first step for diagnostic and vaccine study. This study provides a foundational framework by identifying candidate epitopes that warrant further investigation. Nevertheless, translating these predictions into effective diagnostic tools or vaccine components will require rigorous in vitro and in vivo validation to confirm their immunogenicity, specificity, and relevance across different demographic groups. As such, this work should be viewed as an initial step in a broader, multi-phase research process aimed at the rational design of immunological interventions.

5. No investigation is made into how the predicted epitopes might be processed or presented in the context of actual MHC molecules or host immune systems.

Re: We acknowledge that our study does not investigate the processing or presentation of the predicted epitopes in the context of MHC molecules or host immune systems. Our primary aim was to conduct a structural analysis of Camp Hill virus (CHV) proteins using computational methods, laying the groundwork for future functional and immunological studies. While epitope prediction offers preliminary insights, we agree that experimental validation and deeper exploration of antigen processing and MHC presentation pathways are essential next steps. We hope future work will build on our findings to address these important immunological aspects.

6. The manuscript has several grammatical errors, awkward phrasing (e.g., “CHV is not infected in humans”), and repetitive expressions (e.g., “The predicted structure provides insights…” is repeated verbatim)

Re: Although CHV is not known to infect humans, direct contact or exposure to infected secretions or feces could present a risk.

I have re-written or taken out the text: “The predicted structure provides insights…”

7. Figures and tables are cited inconsistently and sometimes lack proper legends or interpretation.
Re: I have edited the text based on the suggestions.

8. The discussion repeats large portions of the introduction

Re: I have edited the Discussion section.

9. A critical appraisal of results is missing, such as why certain proteins share higher identity with other henipaviruses, and implications for cross-reactivity or zoonotic potential

Re: I have included statements on these in the Discussion section.

Minor comments:-

1. Table 3 should include a clearer explanation of how similarity scores were calculated (e.g., pairwise BLAST or Clustal Omega?)

Re: Pairwise BLAST was used to calculate similarity.

2. Figures 1–8: Consider integrating the B-factor and solvent accessibility plots into a single composite figure per protein.

Re: The figures will appear as a single composite figure in the final version of the manuscript.

3. The Data Availability Statement should specify if model PDB files will be made public
Re: The model PDB files are not made public.

4. Abbreviations should be defined consistently on first use (e.g., RMSD, TM-score). Consider preparing an abbreviation table.

Re: The quality and reliability of the predicted protein model were assessed using structural validation metrics, including the confidence score (C-score), template modeling (TM-score), and root mean square deviation (RMSD). The table is prepared based on the statement. The expansion and abbreviation are stated in the table legend.

**Reviewer #2:** Nipah virus has the potential for recurrent outbreaks and a high fatality rate. Currently, there are no effective preventive vaccines or specific antiviral drugs available, and treatment primarily relies on systemic symptomatic supportive therapy. Nipah virus has been included by the World Health Organization in its Priority Pathogens Blueprint List, necessitating monitoring and preparedness measures to prevent a pandemic.

The paper presents structural analysis and functional predictions of Camp Hill virus (CHV), a newly discovered Henipavirus, first reported in North America. A systematic structural analysis was conducted on the viral attachment glycoprotein, fusion protein, X protein, C protein, matrix protein, nucleocapsid protein, phosphoprotein, and RNA polymerase, focusing on the presence and location of viral proteins, domains and spatial conformation of receptor interactions and immune recognition, B-cell and T-cell epitopes, structural feature analysis, and recognition regions of antibody and immune effector.

The paper is logically clear, methodologically systematic, and conclusive. It provides structural biology reference data for understanding the possibility of human infection with CHV, comprehending its potential transmission routes and those of other shrew-borne viruses, formulating corresponding prevention strategies including vaccines, and identifying potential targets for drug development.

Although a study does not need to be fully reflected in one single paper, virus structure prediction articles, such as this paper, which analyzes from a bioinformatics perspective, do not have any major flaws. However, from a virology perspective, it is still hoped that the corresponding prediction conclusions can be verified in terms of protein function at levels of the cellular or corresponding model animals especially.

Re: The primary objective of this manuscript is to characterize the structural features of the (CHV proteins through comprehensive computational analysis. To our knowledge, this study represents the first in-depth structural investigation of CHV proteins, offering a foundational dataset that can inform and guide future functional, mechanistic, and translational research. While we acknowledge the importance of incorporating comparative and functional insights, particularly those supported by experimental validation, such analyses fall beyond the scope of the current work. Our focus here is to establish a structural framework that can serve as a critical reference point for subsequent empirical studies aiming to explore the biological roles and therapeutic potential of CHV proteins.

In addition, please ensure consistency in the number of decimal places retained for the percentages in Table 3.

Re: I have edited the decimals in the table.
